# The role of histological subtype and chemotherapy on prognosis of ureteral cancer

**DOI:** 10.1007/s00432-024-05684-8

**Published:** 2024-04-13

**Authors:** Jincong Li, Yuxuan Song, Yun Peng, Jiaxing Lin, Yiqing Du, Caipeng Qin, Tao Xu

**Affiliations:** Department of Urology, Peking University People’s Hospital, Beijing, 100044 China

**Keywords:** Ureteral cancer, Histological type, Chemotherapy, T stage, Cancer-specific survival, Overall survival

## Abstract

**Objective:**

To date, there have been few studies examining the prognostic implications of histological subtypes in ureteral cancer. And chemotherapy plays a crucial role in the treatment of ureteral cancer, while many factors influence the efficacy of chemotherapy. This study aimed to utilize the Surveillance, Epidemiology and End Results database to assess the impact of histological type on ureteral cancer prognostic outcomes and discovered how histological type and T-stage influence the efficacy of chemotherapy.

**Methods:**

Based on Surveillance, Epidemiology, and End Results Program, we reviewed 8915 records of patients with primary ureteral cancer from 18 centers between 2000 and 2018. We focused on the overall survival and cancer-specific survival of the records and used Kaplan‒Meier method to calculate survival curves.

**Results:**

In the comparison of prognostic outcomes, atypical subtypes exhibited a less favorable prognosis compared to typical ureteral carcinoma. Notably, patients diagnosed with papillary urothelial carcinoma demonstrated the most favorable overall survival (p = 0.005). Statistically significant benefits were observed in the prognosis of patients with non-papillary urothelial carcinoma who received chemotherapy (HR = 0.860, 95% CI 0.764–0.966, p = 0.011), while chemotherapy did not yield a statistically significant effect on the prognosis of patients with papillary urothelial carcinoma (HR = 1.055, 95% CI 0.906–1.228, p = 0.493). Chemotherapy had an adverse impact on the prognosis of patients with T1 ureteral cancer (HR = 1.235, 95% CI 1.016–1.502, p = 0.034), whereas it exhibited a positive prognostic effect for T3/T4 cases (HR = 0.739, 95% CI 0.654–0.835, p < 0.001).

**Conclusions:**

Histological type affects the prognosis of ureteral cancer. And evaluation of cancer histological type and T stage in ureteral cancer patients prior to chemotherapy is mandatory.

**Supplementary Information:**

The online version contains supplementary material available at 10.1007/s00432-024-05684-8.

## Introduction

Urothelial carcinomas are the sixth most common tumours in developed countries (Siegel et al. 2021). Per the European Association of Urology (EAU) guidelines (Rouprêt et al. 2018) and numerous clinical investigations, renal pelvic cancer (RPC) and ureteral cancer (UC) are considered an integral group and are collectively referred to as upper urinary tract urothelial carcinoma. Nevertheless, clinical and epigenetic disparities suggest that renal pelvic and ureteral tumors may represent distinct disease entities. Recent research indicates that patients diagnosed with ureteral tumors experience an unfavorable prognosis (Rouprêt et al. 2021). Fujii et al. (2021) conducted a comprehensive molecular study of upper urinary tract urothelial carcinoma using unbiased, multiplatform analyses and observed distinctions in the molecular characteristics between RPC and UC. Histological subtypes of tumors at certain sites were reported to be likely to affect the prognosis (Zhou et al. 2022; Beadsmoore and Screaton 2003; Davy et al. 2003). Zhou et al. (2022) reported that prostate cancer with neuroendocrine subtype had the worst survival among all the histological types of prostate cancer. Beadsmoore et al.’s study on lung cancer prognosis (Beadsmoore and Screaton 2003) revealed that patients with small cell subtype had the worst survival. Davy et al. (2003) investigated the prognosis of cervical cancer and noted that adenocarcinoma subtypes were associated with a less favorable prognosis compared to typical squamous cell carcinoma. We suspect that histological type might also affect the prognosis of UC. To date, there have been few studies examining the prognostic implications of histological subtypes in UC. Therefore this study aimed to utilize the Surveillance, Epidemiology, and End Results (SEER) database to assess the impact of histological type on UC prognostic outcomes. Additionally, we investigated the effects of chemotherapy on prognosis.

## Methods

### Data acquisition

Based on SEER, we reviewed 8915 records of patients with primary UC from 18 centers between 2000 and 2018. The records include age at diagnosis, sex, race, Histological Type ICD-O-3, tumor grade, TNM stages of the cancer, survival, data, surgery recode and chemotherapy recode. There are many histological types of UC including papillary urothelial carcinoma, non-papillary urothelial carcinoma, small cell carcinoma, adenocarcinoma, squamous cell carcinoma, papillary carcinoma, spindle cell carcinoma. Urothelial carcinoma is the predominant form of urinary tract cancer (Song et al. 2023). Therefore, we categorized papillary urothelial carcinoma and non-papillary urothelial carcinoma as “typical UC” and classified the remaining histological types as “atypical subtypes”. The quantities of records of some histological types of UC are too low (< = 20) and some histological types are not otherwise specified, so we categorized all of them as “other types of ureter carcinoma”, including large cell carcinoma, pleomorphic carcinoma, pseudosarcomatous carcinoma, combined small cell carcinoma, clear cell adenocarcinoma, renal cell carcinoma and granular cell carcinoma. The classification of all the histological types is shown in Supplementary Table 1.

### Statistics analyses

This study focused on the overall survival (OS) and cancer-specific survival (CSS). OS is the time from diagnosis to death due to any cause, with censoring at the last visit date within SEER or the date of death due to any cause. CSS is the time from diagnosis to death from UC, with censoring at the last visit date within SEER or the date of death due to any other cause. Survival curves were calculated by the Kaplan‒Meier method. Log-rank test was used to compare different survival curves. A multivariable Cox regression analysis was used to identify independent prognostic factors for UC. The chi-square test was used for comparisons between groups. Statistical analyses were performed with IBM SPSS Statistics 26. P values < 0.05 were considered statistically significant.

## Results

### Clinical characteristics of typical UC and atypical subtypes

**Fig. 1:**
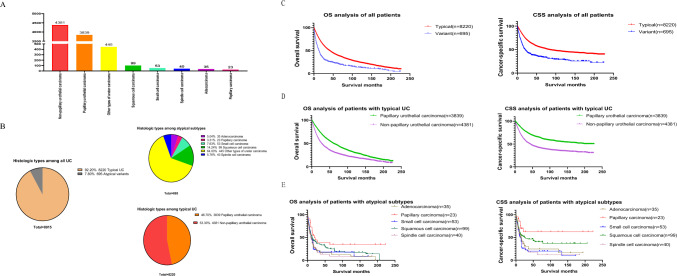
Summary of histological type and prognosis analysis of different groups of histological types. **A** Truncate histogram of histological type statistics **B** Pie chart of histological type statistics **C** Prognosis analysis of all patients **D** Prognosis analysis of patients with typical UC **E** Prognosis analysis of patients with atypical subtype

In total, 8915 patients were enrolled, of which 8220 (92.2%) exhibited typical UC, while 695 (7.8%) presented as atypical subtypes. Clinical parameters of patients with typical carcinoma and atypical subtypes are detailed in Table 1. N-stage and M-stage of atypical subtypes were higher than typical UC (all p < 0.001). Figure 1 presents the statistical data concerning histological types. Non-papillary urothelial carcinoma accounted for 53.30% of cases, while papillary urothelial carcinoma represented 46.70%. Among atypical subtypes, other types of ureter carcinoma was the most prevalent, accounting for 64.03%.

**Table 1:** Clinical characteristics of typical UC and atypical subtypes

Variables	Typical UC	Atypical variant UC	p value
	(n = 8220)	(n = 695)	
Sex (%)			0.001
Female	38	31	
Male	62	69	
Age at diagnosis (%)			< 0.001
1–59	10.2	9.8	
60–74	39.7	33.8	
≥ 75	50.1	56.4	
Race (%)			0.082
White	87.4	86.3	
Black	4.3	5.3	
Other	8.2	7.9	
Unknown	0.1	0.5	
T-stage (%)			< 0.001
T1	33.9	20.3	
T2	21.9	9.1	
T3/T4	27.7	27.5	
Tis	0.1	0	
Unknown	16.3	43.1	
N-stage (%)			< 0.001
N0	80	51.2	
N1	5.3	8.6	
N2	4.7	7.1	
N3	0.5	1.3	
Unknown	9.5	31.8	
M-stage (%)			< 0.001
M0	87.9	60.1	
M1	8.1	20.1	
Unknown	4	19.8	
Grade (%)			< 0.001
High	64.8	35.8	
Low	19.4	9.8	
Unknown	15.8	54.4	
Surgery (%)			< 0.001
No surgery	19.6	20.8	
Local tumor destruction or excision	8.6	9.5	
Segmental resection	32.7	35	
Radical nephroureterectomy	39.1	34.7	
Chemotherapy (%)			0.017
No	79.9	76.1	
Yes	20.1	23.9

### Effects of histological types on prognosis

Kaplan–Meier curves revealed that all atypical subtypes had worse CSS and OS than typical urothelial carcinoma (all p < 0.001; Fig. 1C). In addition, we divided all patients into distinct histological type groups. Kaplan–Meier curves illustrated that papillary urothelial carcinoma had better CSS and OS than non-papillary urothelial carcinoma (all p < 0.001; Fig. 1D). Among all the atypical subtypes, papillary carcinoma demonstrated the most favorable CSS and OS outcomes (CSS: p = 0.05, OS: p = 0.005; Fig. 1E). Multivariate analysis showed histological subtype as an independent risk factor for an unfavorable prognosis (HR = 1.602, 95% CI 1.371–1.872, p < 0.001; Table 2).

**Table 2:** Cox regression analysis evaluating variables associated with overall survival of all UC patients

Variable	Univariate analysis		Multivariate analysis	
	HR (95% CI)	p-value	HR (95% CI)	p-value
Age	1.044 (1.042–1.047)	< 0.001	1.045 (1.042–1.049)	< 0.001
Sex		0.371		< 0.001
Female	Reference		Reference	
Male	0.977 (0.928–1.028)		1.131 (1.059–1.208)	
T-stage		< 0.001		< 0.001
T1	Reference		Reference	
T2	1.071 (0.995–1.153)	0.07	1.060 (0.976–1.151)	0.167
T3/T4	1.916 (1.793–2.047)	< 0.001	1.673 (1.541–1.816)	< 0.001
N-stage		< 0.001		< 0.001
N0	Reference		Reference	
N1	2.091 (1.887–2.318)	< 0.001	1.501 (1.307–1.723)	< 0.001
N2	2.520 (2.257–2.813)	< 0.001	1.555 (1.323–1.827)	< 0.001
N3	2.424 (1.794–3.274)	< 0.001	1.225 (0.762–1.970)	0.402
M-stage		< 0.001		< 0.001
M0	Reference		Reference	
M1	4.461 (4.117–4.833)		2.786 (2.406–3.225)	
Grade		< 0.001		< 0.001
Low	Reference		Reference	
High	1.658 (1.547–1.776)		1.377 (1.265–1.497)	
Chemotherapy		< 0.001		0.121
No	Reference		Reference	
Yes	1.142 (1.072–1.217)		0.931 (0.850–1.019)	
Histological Type		< 0.001		< 0.001
Typical	Reference		Reference	
Variant	1.965 (1.799–2.147)		1.602 (1.371–1.872)	
Surgery Method		0.504		0.526
No surgery	Reference		Reference	
Local tumor destruction or excision	0.929 (0.836–1.031)	0.166	1.022 (0.897–1.164)	0.744
Segmental resection	0.990 (0.921–1.063)	0.778	1.024 (0.935–1.122)	0.609
Radical nephroureterectomy	0.970 (0.905–1.040)	0.396	1.064 (0.974–1.163)	0.166

### Effects of chemotherapy on prognosis of different histological types

**Fig. 2:**
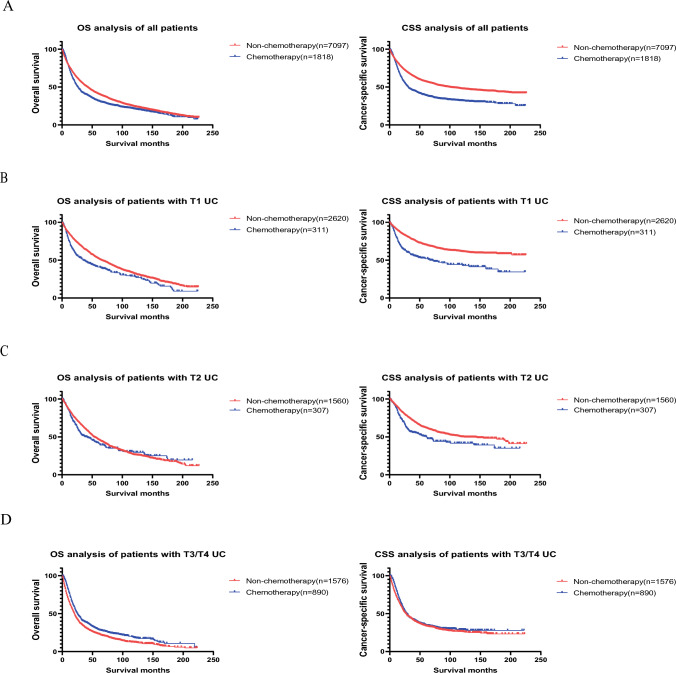
Effects of chemotherapy on prognosis of different histological types **A** Effects of chemotherapy on all patients **B** Effects of chemotherapy on patients with T1 UC **C** Effects of chemotherapy on patients with T2 UC **D** Effects of chemotherapy on patients with T3/T4 UC

We divided all patients into chemotherapy and nonchemotherapy group. Kaplan–Meier curves demonstrated that the non-chemotherapy group exhibited superior OS and CSS (all p < 0.001; Fig. 2A). However, multivariate analysis indicated that chemotherapy had no statistically significant impact on the prognosis of all patients (HR = 0.931, 95% CI 0.850–1.019, p = 0.121; Table 2).

To investigate the impact of chemotherapy on various histological type of UC, we categorized the patients into distinct histological type groups. Records of papillary urothelial carcinoma and non-papillary urothelial carcinoma were sufficient for multivariate Cox analysis, while others (atypical subtypes) were of insufficient quantity. Therefore we divided all the rest of the patients with atypical UC into one group for multivariate Cox analysis. We also constructed Kaplan–Meier curves to evaluate the impact of chemotherapy within each group of atypical subtypes.

Multivariate Cox analysis revealed that chemotherapy had no statistically significant effect on the prognosis of patients with papillary urothelial carcinoma (HR = 1.055, 95% CI 0.906–1.228, p = 0.493; Supplementary Table 3). However, chemotherapy demonstrated a statistically significant positive impact on the prognosis of patients with non-papillary urothelial carcinoma (HR = 0.860, 95% CI 0.764–0.966, p = 0.011; Supplementary Table 2).

The multivariate Cox analysis on all patients with atypical UC indicated that chemotherapy had no statistically significant effect on the prognosis (HR = 0.997, 95% CI 0.675–1.473, p = 0.987; Supplementary Table 4). Chemotherapy group of patients with small cell carcinoma exhibited improved OS and CSS than non-chemotherapy group (OS: p = 0.003, CSS: p = 0.014; Supplementary Fig. 1B). However, Kaplan–Meier curves revealed that chemotherapy group had worse CSS than non-chemotherapy group among patients with squamous cell carcinoma or papillary carcinoma (Squamous cell carcinoma: p = 0.03; Papillary carcinoma: p = 0.002; Supplementary Fig. 1).

### Effects of chemotherapy on prognosis of different T stages

**Fig. 3:**
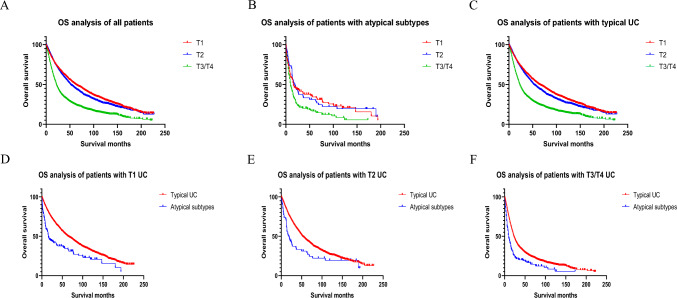
Stratified analysis of T-stage effects on prognosis of different histological types **A** Effects of T-stage on all patients **B** Effects of T-stage on patients with atypical subtypes **C** Effects of T-stage on patients with typical UC **D** Effects of histological types on patients with T1 UC **E** Effects of histological types on patients with T2 UC **F** Effects of histological types on patients with T3/T4 UC

Stratified analysis of T1, T2, T3/T4 was performed for typical UC and atypical subtypes. Patients with lower T-stage were shown to have better prognosis (all p < 0.001; Fig. 3A–C**).** Kaplan–Meier curves revealed that all atypical subtypes had worse CSS and OS than typical urothelial carcinoma among patients with T1 UC, T2 UC, and T3/T4 UC (all p < 0.001; Fig. 3D–F).

Kaplan–Meier curves demonstrated that nonchemotherapy group of patients in T1 group had better OS and CSS (OS: p < 0.001, CSS: p < 0.001; Fig. 2B), while nonchemotherapy group of patients in T3/T4 group exhibited poorer OS and CSS (OS: p < 0.001, CSS: p = 0.026; Fig. 2D). In T2 group, nonchemotherapy group of patients had better CSS while chemotherapy had no statistically significant effect on OS (OS: p = 0.237, CSS: p < 0.001; Fig. 2C).

The multivariate analysis on patients in T1 group revealed that chemotherapy had statistically significant negative effect on prognosis (HR = 1.235, 95% CI 1.016–1.502, p = 0.034; Supplementary Table 5). However, chemotherapy had no statistically significant effect on the prognosis of patients in T2 group (HR = 1.103, 95% CI 0.916–1.329, p = 0.3; Supplementary Table 6). Chemotherapy had statistically significant positive effect on the prognosis of patients in T3/T4 group (HR = 0.739, 95% CI 0.654–0.835, p < 0.001; Supplementary Table 7).

## Discussion

In this research, among all the histological type of UC, patients with typical urothelial carcinoma exhibited superior CSS and OS compared to those with atypical UC. Marina et al. (Deuker et al. 2021) concluded that disease stage at diagnosis is more advanced in upper urinary tract tumors with variant histology patients than pure upper urinary tract urothelial carcinoma. This could potentially result in inferior CSS and OS, aligning with our research findings. Song et al. (2023) reported that patients with upper tract variant histology were more likely to present at advanced stages and experience higher mortality rates when compared to pure UC, which is consistent with our findings.

Retrospective studies based in Korea (Lee et al. 2006) and Japan (Soga et al. 2008) did not identify a benefit from adjuvant chemotherapy. However, a phase 3 multicentre trial evaluating the benefit of four cycles of adjuvant gemcitabine-platinum combination chemotherapy initiated within 90 d after nephroureterectomy versus surveillance reported a significant improvement in disease-free survival (DFS) in patients with upper urinary tract tumors (Birtle et al. 2020). Leow et al. (2021) conducted a meta-analysis investigating neoadjuvant chemotherapy or adjuvant chemotherapy for upper urinary tract tumors and discovered that adjuvant chemotherapy provided a benefit in OS, CSS, and DFS compared with radical nephroureterectomy alone. According to European Association of Urology guidelines (Rouprêt et al. 2023), adjuvant chemotherapy should be contemplated for urothelial carcinoma. Nevertheless, according to Thomas et al (2013), a decline in renal function following radical nephroureterectomy reduces eligibility for cisplatin-based chemotherapy, thereby constraining the use of adjuvant chemotherapy.

While studies focusing on adjuvant chemotherapy have yielded conflicting outcomes, numerous research on neoadjuvant chemotherapy have demonstrated consistent results. Although the use of carboplatin in a neoadjuvant setting remains a subject of debate (Koie et al. 2014; Koie et al. 2013; Dogliotti et al. 2007; Ohyama et al. 2014; Park et al. 2013; Fukushi et al. 2017), cisplatin-based neoadjuvant chemotherapy for patients with UC was exhibited to be safe. And cisplatin-based neoadjuvant chemotherapy is often proposed in patients with metastatic or locally advanced UC (Audenet et al. 2013). Matin et al. (2010) evaluated the incidence of pathologic downstaging and complete remission in patients with high-grade upper urinary tract tumors who received neoadjuvant chemotherapy followed by surgery. And they discovered that neoadjuvant chemotherapy was associated with a 14% complete remission rate and a significant rate of downstaging. Hosogoe et al. (2018) discovered that platinum-based neoadjuvant chemotherapy for advanced upper urinary tract urothelial carcinoma potentially improves oncological outcomes.

Due to limitation of SEER, our research could not differentiate between adjuvant chemotherapy and neoadjuvant chemotherapy. However, our research demonstrated varying effects of chemotherapy on the prognosis of UC with different histological subtypes, which might explain the reason why studies focusing on adjuvant chemotherapy have yielded conflicting outcomes. Apart from histological subtypes, other factors were reported to influence the prognosis of UC. Dabi et al. (2018) found that higher BMI was associated with higher cancer-specific mortality in patients treated with radical nephroureterectomy for upper tract urothelial carcinoma. Van et al. (Osch et al. 2016) discovered that lifetime cigarette smokers were at increased risk for a more malignant type of urothelial carcinoma associated with a worse prognosis. According to Tai et al. (2015), diabetes mellitus with poor glycemic control increases bladder cancer recurrence risk in patients with upper urinary tract urothelial carcinoma. Raj et al. (2006) discovered that patients with involved ureters and/or ureteral anastomotic margins have a higher risk of upper tract recurrence. Inamoto et al. (2020) reported that Patients with lower ureteral tumors had a higher prevalence of deaths compared to patients with upper ureter tumors. Fibroblast growth factor receptor 3 mutation were reported to exacerbate the treatment of UC patients (Song, et al. 2023).

Shinohara et al. (1995) performed clinical investigation of 93 patients with upper urinary tract urothelial carcinoma. They observed that chemotherapy had no impact on survival across all stages compared to the non-chemotherapy group. However only for T3/T4 cases, cisplatin-based chemotherapy improved the prognosis compared with patients without chemotherapy, which is consistent with our findings.

## Conclusion

UC is a clinically important disease that carries a particularly unfavorable prognosis. Among all the histological types, patients with papillary urothelial carcinoma exhibited the most favorable prognosis. Chemotherapy yielded diverse effects across various histological types of UC. Statistically, chemotherapy demonstrated a positive impact on the prognosis of patients with non-papillary urothelial carcinoma, whereas its effect on patients with papillary urothelial carcinoma lacked statistical significance. Different T stages exhibited varying benefits from chemotherapy, with T3/T4 cases potentially benefiting more, while T1/T2 cases may not derive benefits from chemotherapy. According to our results, evaluation of cancer histological type and T stage in UC patients prior to chemotherapy is mandatory.

### Supplementary Information

Below is the link to the electronic supplementary material.

Supplementary file 1: Supplementary table 1. Classification of all the histological types. Supplementary table 2. Cox regression analysis evaluating variables associated with overall survival of patients with non-papillary urothelial carcinoma. Supplementary table 3. Cox regression analysis evaluating variables associated with overall survival of patients with papillary urothelial carcinoma. Supplementary table 4. Cox regression analysis evaluating variables associated with overall survival of patients with atypical UC. Supplementary table 5. Cox regression analysis evaluating variables associated with overall survival of patients with T1 UC. Supplementary table 6. Cox regression analysis evaluating variables associated with overall survival of patients with T2 UC. Supplementary table 7. Cox regression analysis evaluating variables associated with overall survival of patients with T3/T4 UC. Supplementary figure 1. Effects of chemotherapy on prognosis of different histological types (A) Effects of chemotherapy on patients with squamous cell carcinoma (B) Effects of chemotherapy on patients with small cell carcinoma (C) Effects of chemotherapy on patients with spindle cell carcinoma (D) Effects of chemotherapy on patients with adenocarcinoma (E)Effects of chemotherapy on patients with papillary carcinoma

## Data Availability

The dataset used in the present study could be accessed from SEER.
