# Supplementary material for: The role of histological subtype and chemotherapy on prognosis of ureteral cancer
Source: J Cancer Res Clin Oncol. 2024 Apr 13;150(4):192. doi: 10.1007/s00432-024-05684-8 (PMC11015994; doi:10.1007/s00432-024-05684-8)

Supplementary Material

**The Role of Histological Subtype and Chemotherapy on Prognosis of Ureteral Cancer**

Jincong Li^1,#^, Yuxuan Song^1,#^, Yun Peng^1^, Jiaxing Lin^1^, Yiqing Du^1^, Caipeng Qin^1,*^, Tao Xu^1,*^

^1^Department of Urology, Peking University People's Hospital, Beijing 100044, China

*** Correspondence:**

Tao Xu
E-mail: [xutao@pkuph.edu.cn](mailto:xutao@pkuph.edu.cn)

Caipeng Qin

E-mail: fances_wind@yeah.net

**# Li and Song contributed equally to this work.**

**Supplementary table 1.** Classification of all the histological types

|  | Histological type | ICD-O-3 code |
| --- | --- | --- |
| Typical UC | papillary urothelial carcinoma | 8130/3 |
|  | non-papillary urothelial carcinoma | 8120/3 |
| Atypical subtypes | small cell carcinoma | 8041/3 |
|  | adenocarcinoma | 8140/3 |
|  | squamous cell carcinoma | 8070/3 |
|  | papillary carcinoma | 8050/3 |
|  | spindle cell carcinoma | 8122/3 |
|  | large cell carcinoma | 8012/3 |
|  | pleomorphic carcinoma | 8022/3 |
|  | pseudosarcomatous carcinoma | 8033/3 |
|  | combined small cell carcinoma | 8045/3 |
|  | clear cell adenocarcinoma | 8310/3 |
|  | renal cell carcinoma | 8312/3 |
|  | granular cell carcinoma | 8320/3 |

**Supplementary table 2.** Cox regression analysis evaluating variables associated with overall survival of patients with non-papillary urothelial carcinoma

| Variable | Univariate analysis | | Multivariate analysis | |
| --- | --- | --- | --- | --- |
|  | HR(95%CI) | p-value | HR(95%CI) | p-value |
| **Age** | 1.039(1.035-1.043) | <0.001 | 1.039(1.034-1.045) | <0.001 |
| **Sex** |  | 0.888 |  | 0.101 |
| Female | Reference |  | Reference |  |
| Male | 1.005(0.936-1.080) |  | 1.082(0.985-1.189) |  |
| **T-stage** |  | <0.001 |  | <0.001 |
| T1 | Reference |  | Reference |  |
| T2 | 0.916(0.820-1.024) | 0.123 | 0.984(0.865-1.119) | 0.804 |
| T3/T4 | 1.540(1.404-1.689) | <0.001 | 1.564(1.393-1.756) | <0.001 |
| **N-stage** |  | <0.001 |  | <0.001 |
| N0 | Reference |  | Reference |  |
| N1 | 1.504(1.320-1.714) | <0.001 | 1.279(1.078-1.519) | 0.005 |
| N2 | 2.052(1.796-2.346) | <0.001 | 1.537(1.272-1.857) | <0.001 |
| N3 | 1.678(1.155-2.437) | 0.007 | 1.530(0.877-2.670) | 0.134 |
| **M-stage** |  | <0.001 |  | <0.001 |
| M0 | Reference |  | Reference |  |
| M1 | 3.556(3.214-3.934) |  | 2.602(2.181-3.105) |  |
| **Grade** |  | <0.001 |  | <0.001 |
| Low | Reference |  | Reference |  |
| High | 1.596(1.407-1.810) |  | 1.318(1.128-1.538) |  |
| **Chemotherapy** |  | 0.288 |  | 0.011 |
| No/Unknown | Reference |  | Reference |  |
| Yes | 0.957(0.881-1.038) |  | 0.860(0.764-0.966) |  |
| **Surgery Method** |  | 0.722 |  | 0.87 |
| No surgery | Reference |  | Reference |  |
| Local tumor destruction or excision | 0.932(0.807-1.076) | 0.339 | 1.025(0.855-1.229) | 0.789 |
| Segmental resection | 0.968(0.877-1.068) | 0.518 | 0.973(0.855-1.106) | 0.673 |
| Radical nephroureterectomy | 0.953(0.866-1.048) | 0.323 | 1.015(0.897-1.148) | 0.815 |

**Supplementary table 3.** Cox regression analysis evaluating variables associated with overall survival of patients with papillary urothelial carcinoma

| Variable | Univariate analysis | | Multivariate analysis | |
| --- | --- | --- | --- | --- |
|  | HR(95%CI) | p-value | HR(95%CI) | p-value |
| **Age** | 1.055(1.050-1.059) | <0.001 | 1.055(1.049-1.061） | <0.001 |
| **Sex** |  | 0.449 |  | 0.002 |
| Female | Reference |  | Reference |  |
| Male | 0.968(0.891-1.052) |  | 1.171(1.062-1.292) |  |
| **T-stage** |  | <0.001 |  | <0.001 |
| T1 | Reference |  | Reference |  |
| T2 | 1.200(1.081-1.331) | 0.001 | 1.084(0.970-1.211) | 0.153 |
| T3/T4 | 1.979(1.770-2.212) | <0.001 | 1.642(1.443-1.870) | <0.001 |
| **N-stage** |  | <0.001 |  | <0.001 |
| N0 | Reference |  | Reference |  |
| N1 | 3.097(2.503-3.830) | <0.001 | 1.984(1.530-2.572) | <0.001 |
| N2 | 2.943(2.291-3.782) | <0.001 | 1.404(0.975-2.020) | 0.068 |
| N3 | 5.745(2.732-12.079) | <0.001 | 0.263(0.063-1.095) | 0.066 |
| **M-stage** |  | <0.001 |  | <0.001 |
| M0 | Reference |  | Reference |  |
| M1 | 5.949(4.961-7.135) |  | 3.903(2.888-5.274) |  |
| **Grade** |  | <0.001 |  | <0.001 |
| Low | Reference |  | Reference |  |
| High | 1.504(1.374-1.645) |  | 1.346(1.209-1.498) |  |
| **Chemotherapy** |  | <0.001 |  | 0.493 |
| No/Unknown | Reference |  | Reference |  |
| Yes | 1.244(1.102-1.404) |  | 1.055(0.906-1.228) |  |
| **Surgery Method** |  | 0.692 |  | 0.607 |
| No surgery | Reference |  | Reference |  |
| Local tumor destruction or excision | 0.972(0.820-1.153) | 0.748 | 1.014(0.834-1.233) | 0.887 |
| Segmental resection | 1.051(0.934-1.182) | 0.41 | 1.069(0.932-1.227) | 0.338 |
| Radical nephroureterectomy | 1.006(0.897-1.129) | 0.915 | 1.087(0.951-1.243) | 0.221 |

**Supplementary table 4.** Cox regression analysis evaluating variables associated with overall survival of patients with atypical UC

| Variable | Univariate analysis | | Multivariate analysis | |
| --- | --- | --- | --- | --- |
|  | HR(95%CI) | p-value | HR(95%CI) | p-value |
| **Age** | 1.029(1.021-1.037) | <0.001 | 1.027(1.011-1.043) | 0.001 |
| **Sex** |  | 0.495 |  | 0.555 |
| Female | Reference |  | Reference |  |
| Male | 0.943(0.796-1.117) |  | 1.102(0.798-1.523) |  |
| **T-stage** |  | 0.001 |  | 0.35 |
| T1 | Reference |  | Reference |  |
| T2 | 0.903(0.636-1.281) | 0.567 | 0.969(0.594-1.580) | 0.898 |
| T3/T4 | 1.494(1.160-1.926) | 0.002 | 1.260(0.847-1.874) | 0.253 |
| **N-stage** |  | <0.001 |  | 0.09 |
| N0 | Reference |  | Reference |  |
| N1 | 2.015(1.509-2.690) | <0.001 | 1.843(1.130-3.006) | 0.014 |
| N2 | 1.819(1.304-2.538) | <0.001 | 1.084(0.555-2.118) | 0.813 |
| N3 | 1.948(0.961-3.947) | 0.064 | 1.731(0.494-6.066) | 0.391 |
| **M-stage** |  | <0.001 |  | 0.017 |
| M0 | Reference |  | Reference |  |
| M1 | 2.550(2.066-3.148) |  | 1.923(1.122-3.297) |  |
| **Grade** |  | 0.009 |  | 0.645 |
| Low | Reference |  | Reference |  |
| High | 1.517(1.111-2.073) |  | 1.096(0.743-1.614) |  |
| **Chemotherapy** |  | 0.567 |  | 0.987 |
| No/Unknown | Reference |  | Reference |  |
| Yes | 0.944(0.776-1.149) |  | 0.997(0.675-1.473) |  |
| **Surgery Method** |  | 0.125 |  | 0.187 |
| No surgery | Reference |  | Reference |  |
| Local tumor destruction or excision | 0.840(0.595-1.187) | 0.323 | 0.851(0.455-1.592) | 0.613 |
| Segmental resection | 1.075(0.850-1.360) | 0.548 | 1.259(0.806-1.966) | 0.312 |
| Radical nephroureterectomy | 1.204(0.954-1.519) | 0.119 | 1.451(0.947-2.224) | 0.087 |

**Supplementary table 5.** Cox regression analysis evaluating variables associated with overall survival of patients with T1 UC

| Variable | Univariate analysis | | Multivariate analysis | |
| --- | --- | --- | --- | --- |
|  | HR(95%CI) | p-value | HR(95%CI) | p-value |
| **Age** | 1.061(1.056-1.067) | <0.001 | 1.066(1.059-1.072) | <0.001 |
| **Sex** |  | 0.562 |  | <0.001 |
| Female | Reference |  | Reference |  |
| Male | 1.029(0.934-1.133) |  | 1.229(1.097-1.376) |  |
| **N-stage** |  | <0.001 |  | <0.001 |
| N0 | Reference |  | Reference |  |
| N1 | 2.901(2.239-3.759) | <0.001 | 2.431(1.738-3.401) | <0.001 |
| N2 | 2.674(2.017-3.546) | <0.001 | 2.317(1.576-3.406) | <0.001 |
| N3 | 0.655(0.164-2.623) | 0.55 | 0.826(0.203-3.364) | 0.789 |
| **M-stage** |  | <0.001 |  | <0.001 |
| M0 | Reference |  | Reference |  |
| M1 | 5.061(4.162-6.155) |  | 2.808(2.034-3.876) |  |
| **Grade** |  | <0.001 |  | <0.001 |
| Low | Reference |  | Reference |  |
| High | 1.421(1.275-1.585) |  | 1.341(1.196-1.504) |  |
| **Chemotherapy** |  | <0.001 |  | 0.034 |
| No/Unknown | Reference |  | Reference |  |
| Yes | 1.316(1.132-1.528) |  | 1.235(1.016-1.502) |  |
| **Surgery Method** |  | 0.989 |  | 0.432 |
| No surgery | Reference |  | Reference |  |
| Local tumor destruction or excision | 0.973(0.803-1.180) | 0.784 | 0.989(0.792-1.235) | 0.923 |
| Segmental resection | 1.002(0.875-1.148) | 0.977 | 1.066(0.910-1.249) | 0.427 |
| Radical nephroureterectomy | 1.003(0.879-1.144) | 0.963 | 1.116(0.958-1.300) | 0.159 |

**Supplementary table 6.** Cox regression analysis evaluating variables associated with overall survival of patients with T2 UC

| Variable | Univariate analysis | | Multivariate analysis | |
| --- | --- | --- | --- | --- |
|  | HR(95%CI) | p-value | HR(95%CI) | p-value |
| **Age** | 1.043(1.037-1.050) | <0.001 | 1.043(1.036-1.050) | <0.001 |
| **Sex** |  | 0.05 |  | 0.917 |
| Female | Reference |  | Reference |  |
| Male | 0.889(0.790-1.000) |  | 0.993(0.875-1.128) |  |
| **N-stage** |  | <0.001 |  | 0.001 |
| N0 | Reference |  | Reference |  |
| N1 | 2.088(1.570-2.777) | <0.001 | 1.809(1.318-2.483) | <0.001 |
| N2 | 2.002(1.441-2.781) | <0.001 | 1.327(0.905-1.945) | 0.148 |
| N3 | 2.318(1.036-5.188) | 0.041 | 1.970(0.852-4.554) | 0.113 |
| **M-stage** |  | <0.001 |  | <0.001 |
| M0 | Reference |  | Reference |  |
| M1 | 5.058(3.861-6.626) |  | 3.482(2.438-4.973) |  |
| **Grade** |  | <0.001 |  | <0.001 |
| Low | Reference |  | Reference |  |
| High | 1.540(1.320-1.796) |  | 1.471(1.252-1.728) |  |
| **Chemotherapy** |  | 0.349 |  | 0.3 |
| No/Unknown | Reference |  | Reference |  |
| Yes | 1.082(0.918-1.276) |  | 1.103(0.916-1.329) |  |
| **Surgery Method** |  | 0.217 |  | 0.517 |
| No surgery | Reference |  | Reference |  |
| Local tumor destruction or excision | 0.778(0.611-0.992) | 0.042 | 0.867(0.672-1.118) | 0.271 |
| Segmental resection | 0.910(0.772-1.071) | 0.256 | 0.885(0.743-1.053) | 0.169 |
| Radical nephroureterectomy | 0.894(0.763-1.047) | 0.164 | 0.933(0.789-1.104) | 0.421 |

**Supplementary table 7.** Cox regression analysis evaluating variables associated with overall survival of patients with T3/T4 UC

| Variable | Univariate analysis | | Multivariate analysis | |
| --- | --- | --- | --- | --- |
|  | HR(95%CI) | p-value | HR(95%CI) | p-value |
| **Age** | 1.031(1.025-1.036) | <0.001 | 1.027(1.021-1.033) | <0.001 |
| **Sex** |  | 0.234 |  | 0.038 |
| Female | Reference |  | Reference |  |
| Male | 1.060(0.963-1.166) |  | 1.121(1.006-1.248) |  |
| **N-stage** |  | <0.001 |  | <0.001 |
| N0 | Reference |  | Reference |  |
| N1 | 1.371(1.184-1.586) | <0.001 | 1.325(1.117-1.571) | 0.001 |
| N2 | 1.711(1.449-2.021) | <0.001 | 1.447(1.183-1.770) | <0.001 |
| N3 | 1.470(0.865-2.499) | 0.155 | 1.286(0.680-2.433) | 0.439 |
| **M-stage** |  | <0.001 |  | <0.001 |
| M0 | Reference |  | Reference |  |
| M1 | 2.939(2.569-3.362) |  | 2.714(2.257-3.264) |  |
| **Grade** |  | 0.018 |  | 0.048 |
| Low | Reference |  | Reference |  |
| High | 1.248(1.040-1.499) |  | 1.220(1.002-1.486) |  |
| **Chemotherapy** |  | <0.001 |  | <0.001 |
| No/Unknown | Reference |  | Reference |  |
| Yes | 0.738(0.669-0.815) |  | 0.739(0.654-0.835) |  |
| **Surgery Method** |  | 0.185 |  | 0.458 |
| No surgery | Reference |  | Reference |  |
| Local tumor destruction or excision | 1.112(0.925-1.338) | 0.259 | 1.154(0.938-1.420) | 0.175 |
| Segmental resection | 1.079(0.944-1.232) | 0.266 | 1.067(0.921-1.237) | 0.385 |
| Radical nephroureterectomy | 1.152(1.013-1.311) | 0.031 | 1.105(0.958-1.274) | 0.17 |

**Supplementary figure 1.** Effects of chemotherapy on prognosis of different histological types (A) Effects of chemotherapy on patients with squamous cell carcinoma (B) Effects of chemotherapy on patients with small cell carcinoma (C) Effects of chemotherapy on patients with spindle cell carcinoma (D) Effects of chemotherapy on patients with adenocarcinoma (E)

Effects of chemotherapy on patients with papillary carcinoma


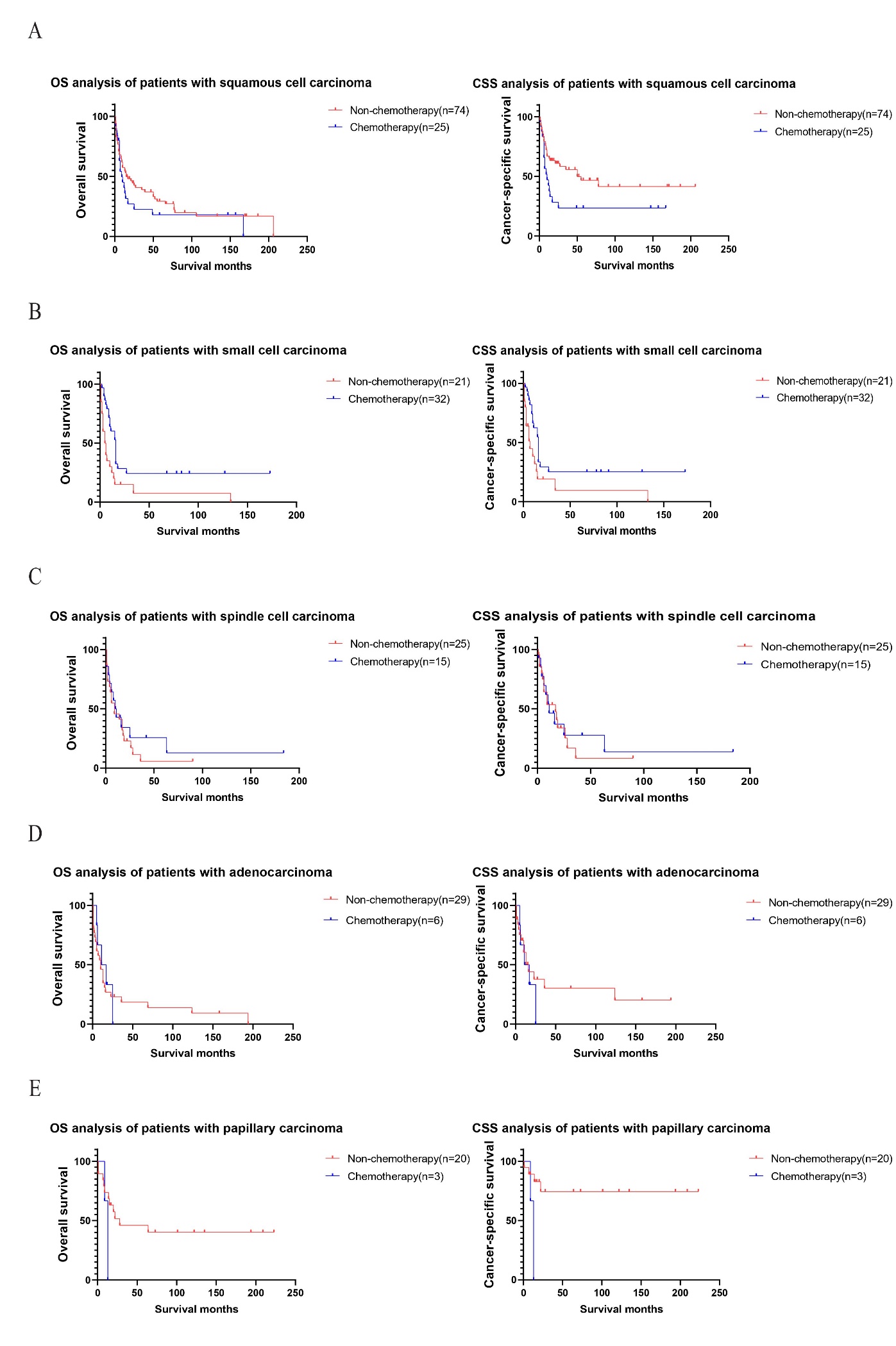

Supplement: Supplementary file 1 — Supplementary table 1. Classification of all the histological types. Supplementary table 2. Cox regression analysis evaluating variables associated with overall survival of patients with non-papillary urothelial carcinoma. Supplementary table 3. Cox regression analysis evaluating variables associated with overall survival of patients with papillary urothelial carcinoma. Supplementary table 4. Cox regression analysis evaluating variables associated with overall survival of patients with atypical UC. Supplementary table 5. Cox regression analysis evaluating variables associated with overall survival of patients with T1 UC. Supplementary table 6. Cox regression analysis evaluating variables associated with overall survival of patients with T2 UC. Supplementary table 7. Cox regression analysis evaluating variables associated with overall survival of patients with T3/T4 UC. Supplementary figure 1. Effects of chemotherapy on prognosis of different histological types (A) Effects of chemotherapy on patients with squamous cell carcinoma (B) Effects of chemotherapy on patients with small cell carcinoma (C) Effects of chemotherapy on patients with spindle cell carcinoma (D) Effects of chemotherapy on patients with adenocarcinoma (E)Effects of chemotherapy on patients with papillary carcinoma [file 432_2024_5684_MOESM1_ESM.docx]
